# Monoamine Release in the Cat Lumbar Spinal Cord during Fictive Locomotion Evoked by the Mesencephalic Locomotor Region

**DOI:** 10.3389/fncir.2017.00059

**Published:** 2017-08-30

**Authors:** Brian R. Noga, Riza P. Turkson, Songtao Xie, Annette Taberner, Alberto Pinzon, Ian D. Hentall

**Affiliations:** The Miami Project to Cure Paralysis, Department of Neurological Surgery, University of Miami Miller School of Medicine Miami, FL, United States

**Keywords:** mesencephalic locomotor region, fictive locomotion, spinal cord, raphespinal, ceruleospinal, monoamine, fast cyclic voltammetry, volume transmission

## Abstract

Spinal cord neurons active during locomotion are innervated by descending axons that release the monoamines serotonin (5-HT) and norepinephrine (NE) and these neurons express monoaminergic receptor subtypes implicated in the control of locomotion. The timing, level and spinal locations of release of these two substances during centrally-generated locomotor activity should therefore be critical to this control. These variables were measured in real time by fast-cyclic voltammetry in the decerebrate cat’s lumbar spinal cord during fictive locomotion, which was evoked by electrical stimulation of the mesencephalic locomotor region (MLR) and registered as integrated activity in bilateral peripheral nerves to hindlimb muscles. Monoamine release was observed in dorsal horn (DH), intermediate zone/ventral horn (IZ/VH) and adjacent white matter (WM) during evoked locomotion. Extracellular peak levels (all sites) increased above baseline by 138 ± 232.5 nM and 35.6 ± 94.4 nM (mean ± SD) for NE and 5-HT, respectively. For both substances, release usually began prior to the onset of locomotion typically earliest in the IZ/VH and peaks were positively correlated with net activity in peripheral nerves. Monoamine levels gradually returned to baseline levels or below at the end of stimulation in most trials. Monoamine oxidase and uptake inhibitors increased the release magnitude, time-to-peak (TTP) and decline-to-baseline. These results demonstrate that spinal monoamine release is modulated on a timescale of seconds, in tandem with centrally-generated locomotion and indicate that MLR-evoked locomotor activity involves concurrent activation of descending monoaminergic and reticulospinal pathways. These gradual changes in space and time of monoamine concentrations high enough to strongly activate various receptors subtypes on locomotor activated neurons further suggest that during MLR-evoked locomotion, monoamine action is, in part, mediated by extrasynaptic neurotransmission in the spinal cord.

## Introduction

The mesencephalic locomotor region (MLR; Shik et al., 1966, 1967) or MLR, a major relay center for the control of locomotion (Garcia-Rill, 1986), activates spinal locomotor generating neurons (Jordan, 1991; Noga et al., 1995a,^2003^) via pathways originating in the medial reticular formation (Orlovskii, 1970; Steeves and Jordan, 1984; Garcia-Rill and Skinner, 1987; Noga et al., 1991, 2003) and descending through the ventral funiculus (Steeves and Jordan, 1980; Noga et al., 1991, 2003). The release of glutamate is thought to play a major role in the activation of these spinal neurons, both in mammals (Douglas et al., 1993; Hägglund et al., 2010; Bretzner and Brownstone, 2013) and in lower vertebrates (Buchanan et al., 1987; Brodin et al., 1989; Ohta and Grillner, 1989).

Monoamines have also been implicated in the activation of spinal locomotor networks. Intravenous injection of L-DOPA, the precursor of dopamine (DA), followed by norepinephrine (NE) produces long-latency, long-duration reflex discharges in acute spinal cats that are qualitatively similar to locomotor movements (Jankowska et al., 1967; Viala et al., 1974). A similar effect, produced by intravenous administration of the serotonin (5-HT) precursor 5-hydroxytryptophan, has been observed in rabbits (Viala and Buser, 1969) and, less reliably, in high spinal cats (Miller et al., 1975). Other monoaminergic drugs likewise evoke or modulate locomotion in spinally injured cats (Barbeau and Rossignol, 1991; Kiehn et al., 1992; Marcoux and Rossignol, 2000) and rats (Feraboli-Lohnherr et al., 1999; Antri et al., 2002) and in *in vitro* neonatal rats (Cazalets et al., 1992; Kiehn and Kjærulff, 1996; Sqalli-Houssaini and Cazalets, 2000) and mice (Christie and Whelan, 2005). Monoamines can be expected to influence locomotion, since terminals of serotonergic and noradrenergic fibers appose spinal locomotor-activated neurons that express a number of monoaminergic receptors implicated in the control of locomotion (Noga et al., 2009, 2011).

Because stimulating the MLR electrically is similar in effect to stimulating the spinal cord with L-DOPA, Grillner and Shik (1973) postulated that the MLR activates a noradrenergic descending pathway, which controls spinal mechanisms for generating locomotion. This idea gained further plausibility when catecholamine-containing cells were found in the vicinity of the MLR (Steeves et al., 1976) and when descending projections from the MLR were found to include the noradrenergic and serotonergic nuclei (Edwards, 1975; Steeves and Jordan, 1984; Sotnichenko, 1985). However, monoamine release is apparently not obligatory since depletion of spinal NE or 5-HT does not abolish the MLR’s ability to evoke locomotion (Steeves et al., 1980).

Nevertheless, there is evidence that monoaminergic pathways are activated during spontaneous or voluntary locomotion. In the cat, the activity of raphespinal and ceruleospinal neurons increases during walking (Fornal et al., 1985, 2006; Rasmussen et al., 1986; Jacobs and Fornal, 1995, 1999; Veasey et al., 1995). A complex pattern of monoamine release has also been observed in the spinal cord of freely moving rats using microdialysis and high performance liquid chromatography (Gerin et al., 1994, 1995, 2008, 2011; Gerin and Privat, 1998).

Based on these findings we hypothesized that MLR stimulation would increase the spinal release of monoamines during evoked locomotion, raising their levels above those observed in resting (basal or steady-state) conditions (Noga et al., 2004). The aim of this study was therefore to determine the extent to which monoamines are released within the spinal cord during MLR-evoked fictive locomotion, to identify the location of this release and its temporal relationship to MLR stimulation and locomotion. The fictive locomotion preparation, in which animals are paralyzed by neuromuscular blockade and locomotor activity is monitored by electroneurogram (ENG) recordings from peripheral nerves, was chosen as the experimental model. This allows investigation of the central drive for induction of locomotion in the absence of peripheral afferent input that by itself can increase spinal release of monoamines (Tyce and Yaksh, 1981; Men et al., 1996). Measurements were made in the gray matter of middle-to-low lumbar segments of the cat where relatively large numbers of serotonergically and noradrenergically innervated locomotor-activated neurons are located (Huang et al., 2000; Dai et al., 2005; Noga et al., 2009, 2011). Measurements were also made in white matter (WM) for comparison to previously obtained microdialysis measurements from the WM of rats subject to treadmill exercise (Gerin et al., 1995). We used fast cyclic voltammetry (FCV; Armstrong-James and Millar, 1984; Stamford et al., 1992) to assess spinal monoamine release by measuring the oxidation of monoamines on the surface of single carbon fiber microelectrodes (CFMEs) during a voltage scan. Individual monoaminergic components of the signal were resolved by Principle Component Regression (PCR; Heien et al., 2004; Keithley et al., 2009). The temporal resolution afforded by this technique contrasts with other extractive methods of measurement, such as microdialysis combined with HPLC, which have temporal resolutions of several minutes and require prolonged conditioning stimulation. The small size of the CFME (33 μm carbon fiber diameter) also allows for higher spatial resolution and is less damaging than the larger microdialysis probes. As the technique measures release relative to a baseline resting state, our experiments were conducted on mesencephalic decerebrate animals in which no spontaneous locomotor activity was observed. Preliminary results have been presented (Noga et al., 2006, 2007).

## Materials and Methods

### Animal Preparation

Experimental procedures were approved by the University of Miami IACUC committee in accordance with National Institute of Health guidelines (NIH Publications No. 80-23; revised 1996). The number of animals used, and their pain and distress, were minimized. Six adult cats (2.8–3.3 kg) were anesthetized with 1%–3% halothane. The trachea was intubated for direct administration of the anesthetic and cannulas inserted into the common carotid artery and jugular vein for blood pressure monitoring and administration of fluids, respectively. Animals were given 2–4 mg of dexamethasone (Hexadrol phosphate, Organon) intravenously to reduce tissue swelling. A bicarbonate solution (100 mM NaHCO_3_ with 5% glucose) was infused at 3–5 ml/h to replace fluid loss and help maintain a normal blood pH. Hindlimb nerves dissected free bilaterally and placed in tunnel electrodes included: semimembranosus/anterior biceps (SMAB), posterior biceps/semitendinosus (PBST), quadriceps (QUAD) and sartorius anterior/medialis (SA). Following a lumbar (L3–6) laminectomy, each animal was placed in a Transvertex headframe and suspended with all limbs pendant. A pool, formed by back muscle and skin flaps, was sealed with a thin layer of Reprosil (Dentsply Caulk, Milford, DE, USA) and filled with warm filtered saline, regularly replaced to prevent accumulation of blood and tissue fluids. Bath and core temperatures were maintained at 37°C using feedback-controlled heating lamps controlled by bath and rectal thermistors. Following a craniotomy, the anesthetic was discontinued and before the animal could awaken a precollicular-postmammillary (mesencephalic) decerebration was performed. After a brief recovery period, the animals were paralyzed with pancuronium bromide (Astra, Westborough, MA, USA: 0.1–0.2 mg/kg as needed—usually every 1–2 h) and artificially ventilated. Ringers solution was sometimes administered intravenously to maintain blood pressure >80 mmHg. Expired CO_2_, O_2_ and tissue oxygenation (SpO_2_) was monitored throughout the experiment using a Datex/Engström Oscaroxy Multigas Monitor and Pulse Oximeter and the end tidal CO_2_ maintained between 3.5% and 4.5%.

### Stimulation and Recording

**Figure 1:**
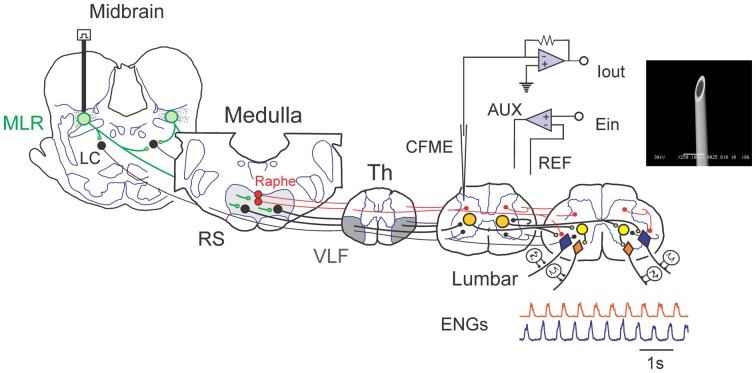
Schematic view of experimental setup used to examine spinal monoamine release during mesencephalic locomotor region (MLR)-evoked fictive locomotion. Known neuronal projections from the MLR include the area of the noradrenergic locus ceruleus (LC), the serotonergic raphe nuclei in addition to the glutamatergic reticulospinal (RS) neurons within the medial reticular formation (Edwards, 1975; Steeves and Jordan, 1984; Sotnichenko, 1985). Axons of RS neurons descend via the ventrolateral funiculus (VLF) to innervate lumbar spinal locomotor interneurons comprising the central pattern generator (CPG; Steeves and Jordan, 1980; Noga et al., 2003). Fast cyclic voltammetry (FCV) scans within the lumbar spinal cord were applied throughout bouts of MLR evoked locomotion. All potentials (Ein) applied to the working carbon fiber microelectrode (CFME) are defined with respect to the reference (REF) electrode. If the potential applied to the CFME is different than the desired potential, then current is provided via the auxiliary (AUX) electrode (Ag/AgCl wire) to maintain the appropriate potential. Fictive locomotor activity is monitored by electroneurogram (ENG) recordings from hindlimb peripheral nerves. *Inset*: scanning electron micrograph of a CFME. The uninsulated carbon fiber (33 μm diameter) is visible at the beveled electrode tip.

The experimental setup is illustrated in Figure 1. Animals were allowed to recover for 1.5–2 h following decerebration before stimulation was commenced. Bouts of locomotion were evoked by electrical stimulation of the MLR (typically 1.0 ms square wave pulses, 15–20 Hz, 30–200 μA) using monopolar stimulating electrodes (SNE-300; David Kopf Instruments, Tujunga, CA, USA). Electrode were stereotaxically inserted into the mesopontine tegmentum at an area bounded by posterior (P) 1–3 and lateral (L) 2.5–5.0 and included the cuneiform nucleus, subcuneiform area, brachium conjunctivum and pedunculopontine nucleus. Electrodes were advanced slowly while stimulating until the optimal response was obtained. If no response or stimulation strength is high, the electrode was repositioned and the procedure repeated. Final position was selected based upon best response and lowest threshold at the specified frequency (15–20 Hz) and pulse width (1.0 ms square wave pulses). Optimal responses were observed with stereotaxic coordinates within the cuneiform and subcuneiform area dorsal to the brachium conjunctivum. Rhythmic activity observed from ENG recordings from hindlimb peripheral nerves, captured digitally as continuous waveforms, was used as an indicator of “fictive” locomotion. ENG signals were amplified with AC-coupled amplifiers (bandwidth 300 Hz–10 kHz), rectified and low-pass filtered (30 ms time constant) and digitized directly through a 1 MHz, 16 channel analog-to-digital converter (12 bit) at 2 kHz, using customized software (Spinal Cord Research Centre, University of Manitoba, Canada).

### Fast Cyclic Voltammetry

Microelectrodes were constructed from single carbon fibers, 33-μm in diameter (Textron Systems, Lowell, MA, USA) inserted into pulled borosilicate capillary tubes (ID 1.12 mm, OD 2.0 mm, WPI, Sarasota, FL, USA) according to procedures described previously (Hentall et al., 2003, 2006; Noga et al., 2004; Brumley et al., 2007). The electrodes were beveled at a 30° or 45° angle from horizontal (Model BV-10 Micropipette Beveller, Sutter Instrument Co., Novato, CA, USA) to produce a sensing elliptical surface defined by the cross section of the carbon fiber, so that the carbon fiber surface was flush with the tip (Figure 1, inset). The electrodes were cleaned in ethyl alcohol and rinsed with de-ionized water. Just prior to use, the electrodes were electrochemically pretreated in phosphate-buffered saline (PBS: 10 ml, pH 7.4) to increase their sensitivity (Stamford et al., 1992) by applying an offset 71 Hz triangular waveform for three consecutive 10-s periods: at −0.6 to +3.0 V, −0.6 to +2.0 V and −0.6 to +1.0 V (Hentall et al., 2003; Noga et al., 2004). In experiments with prolonged scanning and application of metabolic and uptake inhibitors, electrodes were coated with 5% Nafion (Aldrich, St. Louis, MO, USA; dipped five times, then placed in an 80°C oven for 10 min) to improve selectivity for the primary amines and reduce sensitivity to monoaminergic metabolites and ascorbic acid (Brazell et al., 1987).

**Figure 2:**
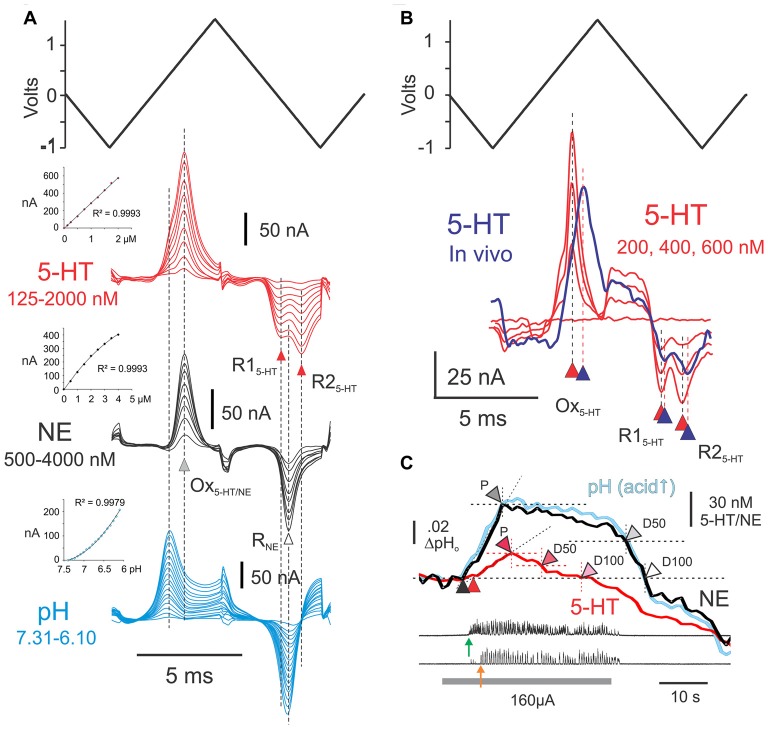
Experimental procedures for detection and estimation of transmitter levels. **(A)** Post-experimental *ex vivo* calibration of CFMEs. Results of *ex vivo* calibrations in different concentrations of 5-HT and norepinephrine (NE) in phosphate buffered saline, pH 7.4 (5-HT: 125, 250, 500, 750, 1000, 1250, 1500, 1750 and 2000 nM; NE: 0.5, 1.0, 1.5, 2.0, 2.5, 3.0, 3.5 and 4.0 μM). Subtracted voltammograms are averages of 40 scans. Also shown is the triangular input waveform (*top*). Peak oxidation currents for 5-HT and NE (Ox_5-HT/NE_) occur at approximately the same voltage *ex vivo* (*gray arrowhead)*. Double (R1_5-HT_ and R2_5-HT_) and single (R_NE_) reduction peaks are observed for 5-HT and NE (*black and white arrowheads*, respectively). The single noradrenergic reduction peak occurs approximately mid-way between the double serotonergic reduction peaks. Subtracted voltammograms illustrating pH effects over the range 7.31–6.1, generated by the addition of concentrated hydrochloric acid (17 steps), are shown beneath the monoamine calibrations. Insets: calibration curves for 5-HT, NE and pH. **(B)** Subtracted voltammogram (*purple line*) obtained from the L6 spinal segment (lamina VII) following microinjection of 100 μM 5-HT near the microelectrode. The voltammogram is an average of 20 scans obtained at the peak of the 5-HT concentration wave observed after injection. It is overlayed with the voltammograms (*red lines*: 20 scans each) obtained during *ex vivo* calibration in concentrations of 5-HT that are within the range of the *in vivo* signal. Note slight delay in peak oxidation and reduction currents. **(C)** Measurements made from principle component regression (PCR) derived *in vivo* signals during MLR evoked locomotor activity. Onset of bursting and locomotor activity are indicated with *green* and *orange arrows*, respectively. *Black* and *red arrowheads* indicate onset of release of NE and 5-HT relative to baseline (pre-locomotor) levels. Other arrowheads indicate peak levels (P), and 50% (D50) and and 100% (D100) decline-to-baseline. MLR stimulation indicated by *gray bar*.

FCV scans were generated using a three-lead voltage-clamp amplifier (Millar Voltammeter, P.D. Systems International Ltd., UK). The leads were connected to the carbon-fiber working electrode, to a carbon-based reference electrode internally filled with KCl (Dri-Ref: WPI, Sarasota, FL, USA) and to an Ag-AgCl auxiliary electrode that applied the clamping current. In a PBS-filled beaker, a triangular voltage waveform (scan) of 14.2 ms duration was applied to the CFME every 0.25 s, sweeping at 480 V/s, from 0 V to −1 V, then to +1.4 V, to −1 V and then back to 0 V (Figure 2, top trace). The amplifier’s output signal, or voltammogram, is proportional to the current flowing through the microelectrode; it was captured digitally at 5 or 10 kHz and calibrated using a 10 or 100 nA current pulse. This output constitutes the so-called “full” signal, which includes the electrode background charging current generated in PBS (or when *in vivo*, in the extracellular fluid of the spinal cord) as well as the much smaller redox current or Faradic current resulting from the oxidation and reduction of any analytes in solution (Brumley et al., 2007). To monitor monoamine concentrations during each experiment, the output voltammogram for each electrode obtained during the first scan in the test sequence was subtracted from subsequent ouput voltammograms to yield a “subtracted voltammogram” of changes in the redox current (Figure 2).

### Electrode Calibration

Electrodes were calibrated after each experiment, in varying concentrations of NE and 5-HT dissolved in PBS at pH 7.4 and at various pH levels. Electrodes are able to measure changes in monoamines from one scan to the next and detection times *in vitro* are likely related to the time required for the solute to reach the electrode (mixing time) with the addition of monoamines to the PBS. Arepresentative calibration set (training set) is illustrated in Figure 2. It is made up of 34 total concentration steps (9 NE, 9 5-HT and 17 for pH; each set done separately), ranging between 250 nM and 4.0 μM for NE, 125–2000 nM for 5-HT and ~7.3–6.1 pH units, respectively. In the presence of monoamines, oxidation currents were generated during the first ascending phase of the triangular input waveform. Oxidation peaks of NE and 5-HT differed only slightly and were maximal when the applied input waveform was near 842 and 827 mV, respectively. The monoamines could be distinguished from each other, however, by the shape of their post-oxidation reduction current(s) produced during the second descending slope of the applied waveform. These smaller currents reflect reduction of the electrode oxidation reaction products that remain nearby (Stamford, 1989). As seen in Figure 2, 5-HT showed double reduction peaks, one near −80 mV and the other near −632 mV, whereas NE showed a single reduction peak near −345 mV. Changes in pH, expected to occur with stimulation *in vivo* (Syková and Svoboda, 1990), induced redox currents that peaked around 450 mV and −300 mV as acid levels increased. Current responses to changes in concentration of monoamines were linear over the range observed *in vivo*. Current responses to changes in pH in the range examined were curvi-linear.

The amplitude of the peak oxidation current was quantified by subtracting a reference level current at one or two points in the subtracted voltammogram where the sign of its slope changed (Hentall et al., 2003, 2006). Concentration changes *in vivo* could then be estimated from peak oxidation currents obtained during electrode calibrations. The mean sensitivity (± SD) of the microelectrodes was 15.2 ± 8.9 (*n* = 6) and 7.0 ± 4.7 (*n* = 6) nM/nA for NE and 5-HT, respectively. Since recording noise for individual scans was ~0.5 nA *in vivo*, detection thresholds were ~1.0 nA, which translates to about 5–10 nM for 5-HT and 10–20 nM for NE (see Hentall et al., 2006).

### *In Vivo* Voltammetry

Monoamine levels were determined at points along microelectrode trajectories within the L4–7 lumbar spinal segments during MLR evoked fictive locomotion. CFMEs were typically inserted into the cord at a 10° (tip rostral) angle. Measurements were usually made every 250 μm up to a depth of ~4500 μm, as measured by the digital readout of the stepping motor that controlled the micromanipulator. Scans were performed at intervals of 250 ms, prior to, during and following 20–60 s trials of MLR stimulation. Monitoring continued for approximately 20–80 s from termination of stimulation. To monitor release during the experiment, subtracted voltammograms were obtained by subtracting the full signal of a pre-locomotion scan, as described for *ex vivo* calibration, above. Subtracted voltammograms obtained *in vivo* are often associated with a linear amplitude change across the full signal (Phillips et al., 2002; Brumley et al., 2007) likely due to changes in tissue impedance during strong neuronal activity. These changes were mostly removed in subsequent offline analysis as described previously (Brumley et al., 2007). To improve the signal-to-noise ratio, single scans obtained at 4 Hz were subsequently averaged to generate an overall 1 Hz sampling frequency prior to processing the data with PCR (Keithley et al., 2009). PCR analysis of the data (Heien et al., 2004) was used to resolve individual components of the voltammograms obtained *in vivo*. To determine concentrations within spinal tissue of three predicted components (NE, 5-HT and pH), a calibration set was generated after each experiment (see *Ex vivo* calibration, above). The number of principal components needed to describe the dataset was between 4 and 6, with the scan range used for this analysis: −1.0 to 1.4 to −1.0 V, as determined using singular value decomposition and a log Scree graph (Keithley et al., 2009). A data matrix was constructed and a regression matrix relating the data projections to concentrations was calculated. PCR accuracy was examined using mixed analyte samples following individual calibrations and was found to have less than 5% error for NE and a 20% error with 5-HT. The shape and value of peak oxidation or reduction potentials depend theoretically on many factors, such as diffusion rates, pH, temperature and redox environment, which can be expected to differ *in vivo* and *ex vivo* (Stamford, 1986; Palij and Stamford, 1994; O’Neill et al., 1998). Peak currents are known to be delayed (shift toward higher scan voltages) with more acidic conditions (Kawagoe et al., 1993), which are found in the spinal gray matter at rest and during periods of electrical stimulation (Syková and Svoboda, 1990; see also Chesler and Kaila, 1992; Chesler, 2003). This expected delay was confirmed by either spinal microinjection or superfusion of NE and 5-HT. These experiments revealed slight delays in redox peaks *in vivo* for 5-HT (see Figure 2B). While such delays may also be due to adsorption of 5-HT to the electrode surface (e.g., Dankoski and Wightman, 2013), electrode responses during experiments were stable and repeatable. Although alternative scanning waveforms are available to reduce such effects, they are too short to reveal double reduction peaks for 5-HT which allow differentiation of 5-HT from catecholamine signals in tissues with mixed monoamine terminal fields. Calibration set peak values were therefore adjusted to compensate for these delays according to subtracted traces from each experiment prior to PCR analysis of *in vivo* data. Residual analysis sometimes revealed additional signals in the dataset that could not be accounted for by the use of three principal components. However, this residual signal fell well outside of the monoaminergic or pH subtracted voltammograms.

Locomotion trials were performed with at least 2 min between trials to allow for clearance and to re-establish monoamine baseline levels when electrodes were advanced to new depths. Repeated trials were done in some locations where large signals were observed, to verify the reproducibility of the measured release and to examine the relationship between monoamine levels and quality of locomotion. The MLR stimulation was not synchronized to the voltammetric scans and any scans containing stimulus artifacts were excluded from the analysis.

In three animals, FCV scans during evoked locomotion were made after the microinjection of the monoamine oxidase inhibitor pargyline and the monoamine uptake inhibitors imipramine, desipramine and buproprion (Rivot et al., 1983; Martin et al., 1988) mixed together in aCSF (2.5–5 μL total volume; in 1 or 2 tracks). This was done to test the effects on monoamine release *in vivo* by blocking the clearance of monamines by uptake mechanisms and metabolism. Microinjections were made using a Neurophore BH-2 pressure ejection system (Medical Systems Corp., Greenvale, NY, USA) through pulled glass micropipettes of ~2–5 μm tip diameter placed 2–3 mm away from the recording electrodes. Micropipettes were initially lowered to the base of the ventral horn (VH; deepest point ~5 mm), and the drugs injected while withdrawing the electrodes toward the surface. Drugs were injected at a rate of 0.5 μL/min and a volume of 0.5–1.0 μL/mm depth (total injection of 5 uL over a ~5 min period). Trials were also conducted during the nearby dialysis of 0.5 mM pargyline and 0.1 mM desipramine in aCSF, from two dialysis probes placed 9–15 mm apart. Electrodes were placed between the probes (4–9 mm distant) and scans conducted during the dialysis.

### Histology

At the end of each experiment, the spinal cord was removed and immersion fixed in 10% formalin. Frozen transverse sections of the cord (100 μm thick) were counterstained with Toluidine blue or Cresyl Violet. Recording sites along reconstructed tracks were determined from microelectrode depth readings (from surface of spinal cord), and taking into account a shrinkage factor for processed tissue (indicated by decreased distances between parallel tracks) and a small correction factor (2%) to account for the 10° (tip-pointing rostral) electrode insertion angle.

### Data Analysis

Locomotor quality was graded using a 0–2 point system for each hindlimb (maximum 4 for bilateral locomotion): 0, no locomotion or tonic activity only; 1, rhythmic excitatory bursts in flexor or extensor nerves without reciprocal rhythmic activity in antagonists; 2, locomotion—full reciprocal alternation between flexors and extensors. Detailed analyses of locomotor activity in some trials included the onset and offset of each step cycle in the recorded ENG bursts, as determined using a threshold detection algorithm with trigger hysteresis. The locomotor frequency, burst durations, duty cycle (proportion of cycle period in which the nerve is active during the step cycle) and burst areas were calculated. Changes in 5-HT and NE concentration and pH during and following MLR stimulation were plotted relative to baseline (pre-stimulation) values. A number of measurements were made from these plots (Figure 2C) and included: (1) onset and offset of bursting and/or locomotor activity relative to stimulation onset; (2) onset and offset latencies of 5-HT/NE concentration and pH changes; and (3) the time-to-peak (TTP) and the time for 50% and 100% decay to baseline (D_50_ and D_100_). To determine onset of stimulated release we visually determined the point where the slopes of the signal at baseline and during stimulation intersected. Profiles of release were categorized. For repeated trials in the same location, cycle statistics for the best continuous bout of locomotion included the frequency and area of the averaged step cycle. Circular statistics (Liu and Jordan, 2005), were used to determine coordination of flexor and extensor nerve activity from ENG recordings on the same and opposite sides. Cycle onsets and offsets were first marked for each nerve. Phase values (Φ) for each step cycle were calculated by dividing the latency between the onsets of paired nerve cycles by the step cycle period. These were then displayed graphically as data points on a polar plot where a phase values of 180° represents out-of-phase activity (alternation) and values of 0° or 360° are in-phase and equivalent to each other. The mean phase value is indicated by the direction of a vector, with length *r* (Zar, 1974). The length of the vector ranged from 0 to 1, and is a measure of the concentration of phase lags around the mean phase value. Values of *r* greater than the critical Rayleigh’s circular statistical test value (Zar, 1974) indicated by dotted circles inside of the polar plot (*p* < 0.05 and < 0.01) were considered phase-related.

### Statistical Analysis

Measurement sites were assigned to cytoarchitectural laminae (Rexed, 1954) and various measured parameters were averaged across tracks and subjects. Statistical significance was assessed by independent samples *t*-tests and paired samples *t*-tests to test various hypotheses. Non-parametric tests were used to calculate differences between dichotomous variables. The extent to which various factors would predict quality of locomotion was assessed with linear regression models. All statistical analyses were performed with SPSS 22 for Windows. A level of *p* ≤ 0.05 was considered statistically significant. Mean and SD are reported throughout, unless otherwise indicated.

## Results

### Locomotor Responses to MLR Stimulation

A total of 132 trials of MLR stimulation were conducted for general data analysis of monoamine release from four animals and data from all animals are grouped together unless otherwise stated. Rhythmic excitatory or full locomotor activity was induced in 125 of these trials, with the remaining trials showing only increased tonic nerve activity, bilaterally. Bilateral rhythmicity/locomotion was induced in 120 of 125 trials, with the remaining five trials showing only one-sided locomotion: four with locomotion ipsilateral to the CFME recording electrode and 1 with contralateral locomotion. Latencies from the start of stimulation to the onset of ipsilateral and contralateral locomotion were 16.1 ± 11.8 and 16.8 ± 13.1 s (mean ± SD), respectively, with a range of 2.7–52.5 s. Longer locomotor onset latencies were observed toward the end of the experiments in two animals and in one animal from the beginning. Locomotion could be preceded by the appearance of tonic bursting activity as is seen in other studies of MLR-evoked fictive locomotion (e.g., Noga et al., 1995a; MacDonell et al., 2015).

### Spatial Patterns of Spinal Monoamine Release with MLR Stimulation

**Figure 3:**
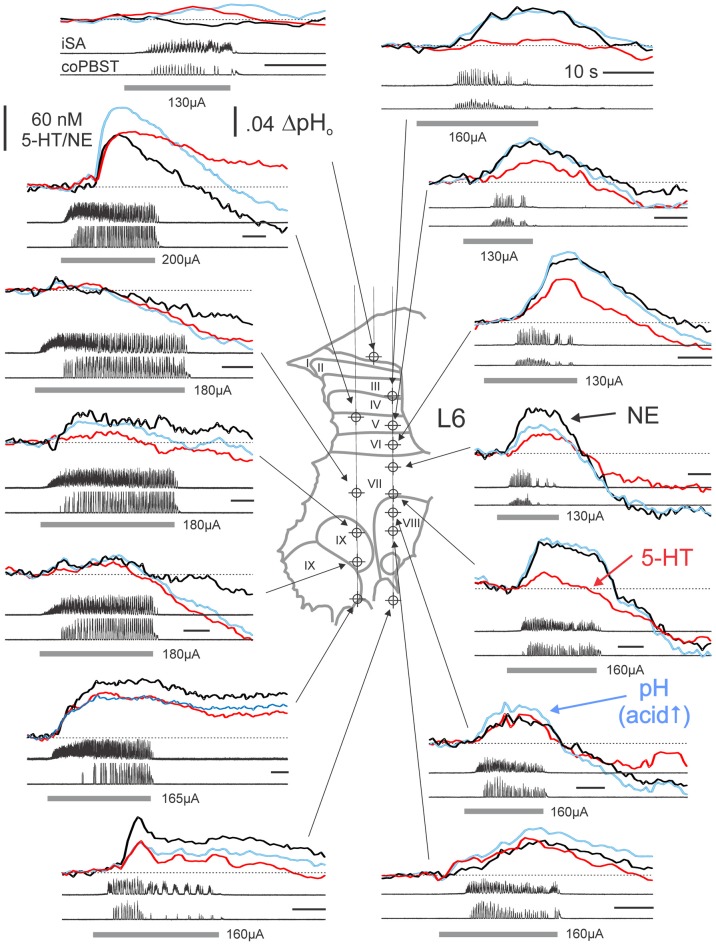
Monoamine release profiles generated at various locations within the L6 spinal segment from a single experiment. Monoamine release profiles determined from PCR analysis of subtracted voltammograms are plotted with respect to the time of occurrence during separate trials of MLR-evoked fictive locomotion. Monoamine concentrations and pH measures reflect relative changes from baseline, non-locomotor (at rest) levels obtained at each site immediately prior to each trial. NE, 5-HT and pH signals are indicated by thick *black*, *red* and *blue* lines, respectively. Recording sites indicated on histological reconstruction of the electrode tracks observed in spinal sections create a “map” of monoamine release. Locomotor activity is observed in ENG recordings from single ipsilateral and contralateral nerves for each trial of MLR-evoked fictive locomotion. *Gray bars* indicate the period of MLR stimulation (ipsilateral to FCV recording; 130–200 μA, 15 Hz, 1 ms duration; strength indicated for each trial). Calibration bars: 10 s. SA: sartorius; PBST: posterior biceps/semitendinosus; i: ipsilateral; co: contralateral.

**Figure 4:**
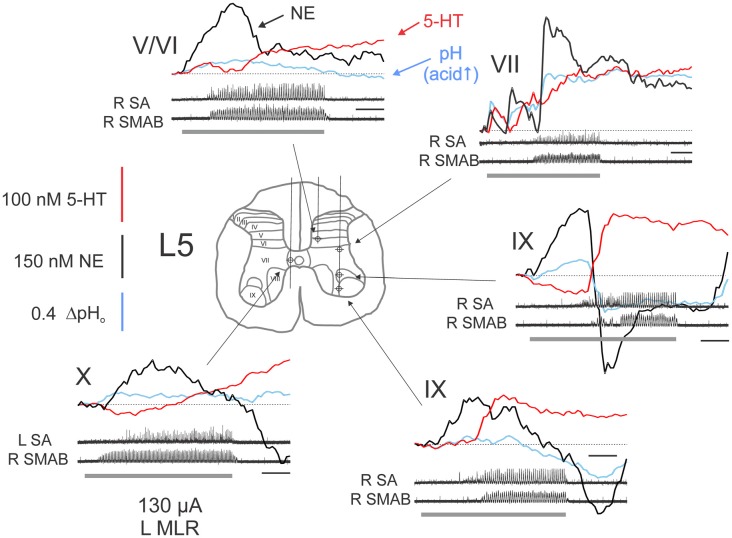
Complex monoamine release profiles observed during trials with delayed MLR-evoked locomotion. Profiles recorded at various locations within the L5 spinal segment from a single experiment. *Gray bars* indicate the period of MLR stimulation (left side, 130 μA throughout, 20 Hz, 1 ms duration). Locomotor activity is indicated by ENG recordings from right (R) and left (L) nerves. Calibration bars: 10 s. SMAB: semimembranosus/anterior biceps. Figure format and abbreviations as in Figure 3.

**Figure 5:**
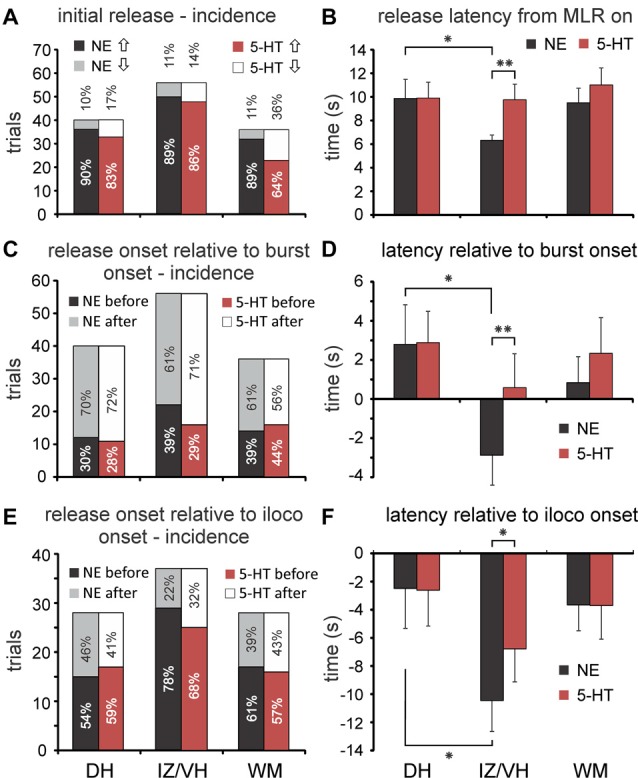
Incidence and latencies of monoamine transmitter release during MLR-evoked fictive locomotion. **(A)** Profiles of initial changes in 5-HT and NE concentrations (incidence) in dorsal horn (DH), intermediate zone/ventral horn (IZ/VH) and in the adjacent white matter (WM) of middle and caudal lumbar segments with stimulation of the MLR. **(B)** Onset latencies of monoamine release (mean and SEM in **B**,**D**,**F**) relative to onset of MLR stimulation. NE onset latencies in the IZ/VH were significantly shorter compared to those observed in the DH and for those observed for 5-HT in the IZ/VH. **(C)** Timing of changes in release in DH, IZ/VH, and WM relative to the onset of burst activity in hindlimb ENGs. Percentages of occurrence (incidences) are given. **(D)** Onset latencies of monoamine release relative to onset of burst activity. NE onset latencies in the IZ/VH were significantly earlier compared to those observed in the DH and for those observed for 5-HT in the IZ/VH. **(E)** Timing of changes in release in DH, IZ/VH, and WM relative to the onset of ipsilateral locomotion. Percentages of occurrence (incidences) are given. Note that in all locations, changes in monoamine release usually occurred before the onset of locomotion. **(F)** Onset latencies of monoamine release relative to onset of ipsilateral locomotion. NE onset latencies in the IZ/VH were significantly earlier compared to those observed in the DH and for those observed for 5-HT in the IZ/VH. *p < 0.05, **p < 0.01.

Changes in extracellular concentrations of monoamines were measured at various locations throughout gray and WM in L4–L6 lumbar segments during MLR evoked fictive locomotion (Figures 3, 4). Levels were measured relative to baseline levels which reflect the dynamic equilibrium between release and uptake during non-locomotor steady state resting conditions. Transmitter profiles most typically observed within the dorsal horn (DH), intermediate zone (IZ)/VH and adjacent WM during evoked locomotion are illustrated in Figure 3. The first major response to MLR stimulation was most commonly an increase in release. This profile was observed in 89.4% and 78.1% of all trials (all locations) for NE and 5-HT, respectively. The MLR-evoked increase in NE or 5-HT levels gradually declined once stimulation was terminated, or before, if locomotor activity diminished during stimulation. In remaining trials, monoamine levels decreased below resting levels with stimulation. Such profiles of release were highly localized since neighboring areas mostly showed increases in transmitter release in subsequent trials. The profile of release (relative incidence of increased or decreased release with stimulation) differed significantly between NE and 5-HT (McNemar test; *p* = 0.035). An initial release of NE occurred in 90.0%, 89.3% and 88.9% of trials in DH, IZ/VH and adjacent WM, with no significant difference between locations (Figure 5A). The relative frequency of increased release of 5-HT in DH, IZ/VH and adjacent WM was 82.5%, 85.7% and 63.9%, respectively; these locations differed significantly (*p* = 0.035; Pearson Chi-Square test).

The extracellular levels of NE and 5-HT could vary in a complex way relative to each other during MLR stimulation (Figure 4), however, and especially if the induction of locomotor activity was somewhat delayed. For example, in lamina IX, the (delayed) onset of locomotor activity was associated with an offset of NE release and the onset of 5-HT release in lamina IX. This pattern of 5-HT release within lamina IX observed in this example indicates that 5-HT release covaries with motoneuron depolarization. This was further corroborated in other recordings from lamina IX. The timing of 5-HT release in lamina IX was related to onset of motoneuron discharge (bursting or locomotion) in all of the nine recorded trials with stable baselines (five examples are shown in Figures 3, 4). Although there are differences in the total amount released from trial to trial, this seems to be related to the length of time for locomotor activity to be initiated. The increase was largest (45–200 nM) in trials with delayed locomotion (>14 s; 6/9 trials) and smallest (16–18 nM) in trials with faster initiated locomotion (≤10 s; 3/9 trials). In contrast, in the lateral portion of lamina VII, the onset of locomotor activity was associated with stabilization and ultimately with dramatic increases in both NE and 5-HT when locomotion was finally observed. Other complex waveforms are illustrated in Figure 4. In lamina X, the initial decrease in extracellular 5-HT with MLR stimulation was reversed with the appearance of ipsilateral locomotor activity. In the same trial, NE release occurred shortly after commencement of MLR stimulation and prior to the onset of ipsilateral locomotor activity. In another example, NE increased after MLR stimulation and dropped to a lower and eventually more stable level as 5-HT levels increased in the DH (laminae V/VI).

### Temporal Pattern of Spinal Monoamine Release with MLR Stimulation

Onset latencies for initial changes in NE or 5-HT level were similar for trials showing increased or decreased release and were therefore grouped together.

#### Latency Relative to Onset of MLR Stimulation

Overall, changes in the extracellular concentration of NE (NEonset_MLR_) and 5-HT (5-HTonset_MLR_) began 8.3 ± 7.3 s and 10.1 ± 9.1 s (mean ± SD; unless otherwise noted) after the start of MLR stimulation. There was a significant difference between locations in DH, IZ/VH and adjacent WM for NEonset_MLR_ latencies (one-way analysis of variance (ANOVA); *F* = 3.544, *p* = 0.032). *Post hoc* testing showed NEonset_MLR_ latency to be significantly shorter (*p* = 0.05, Tukey test) in the IZ/VH (6.3 ± 3.3 s, *n* = 56) than in the DH (9.9 ± 10.3 s, *n* = 40; Figure 5B) but not in the WM (9.5 ± 7.5 s, *n* = 36). 5-HTonset_MLR_ latencies in the DH, IZ/VH and WM (9.9 ± 8.5 s, 9.8 ± 9.9 s, 11.0 ± 8.7 s, respectively) did not show significant differences by ANOVA. Since we observed delays in the onset of 5-HT release in some locations within the IZ/VH (e.g., lamina IX—Figure 4), we did a paired samples *t*-test comparing NEonset_MLR_ latency and 5-HTonset_MLR_ latency in the IZ/VH. The IZ/VH onset latency for NE was significantly shorter (*p* = 0.010, *t* = −2.665).

#### Latency Relative to Onset of Bursting Activity

MLR stimulation could evoke tonic activity prior to the appearance of locomotion in the ENGs (Figure 3). In this analysis, we measured the latency of release relative to the appearance of any kind of ENG activity (tonic or phasic). Overall, MLR-evoked changes in the extracellular concentrations of NE and 5-HT over baseline levels began before the onset of bursting activity (NEonset_burst_ and 5-HTonset_burst_) in 36.4% and 32.6% of trials, respectively. The frequency of occurrence did not statistically differ for recording trials obtained within the DH, IZ/VH and adjacent WM for either NE or 5-HT (Figure 5C) and there were no statistical differences in the frequency of occurrence between NE and 5-HT. However, NEonset_burst_ latencies observed in DH, IZ/VH and WM, revealed an overall significant difference between locations (one-way ANOVA; *F* = 3.156, *p* = 0.046). With *post hoc* testing (Tukey), this was attributable to a significant difference (*p* = 0.043) in the NEonset_burst_ latency in the IZ/VH (−2.9 ± 11.7 s; *n* = 56) compared to the DH, indicating that NE release in the IZ/VH precedes the onset of burst activity whereas in the DH, NE release was observed to occur after the onset of burst activity (2.8 ± 12.8 s; *n* = 40; Figure 5D). No significant difference was noted with *post hoc* testing for NEonset_burst_ latency in the IZ/VH or DH compared to the adjacent WM (0.8 ± 8.0 s; *n* = 36). The overall ANOVA did not show significant differences in 5-HTonset_burst_ latencies in the DH, IZ/VH and adjacent WM (2.8 ± 10.2 s, 0.6 ± 13.0 s and 2.3 ± 10.9 s, respectively). However, a paired samples *t-test* comparing NEonset_burst_ and 5-HTonset_burst_ latency in the IZ/VH revealed that NE release measured relative to the burst onset, was significantly earlier than 5-HT (*p* = 0.010, *t* = −2.665).

#### Latency Relative to Onset of Ipsilateral Locomotion

Overall, MLR-evoked changes in the extracellular concentrations of NE and 5-HT over baseline levels began before the onset of ipsilateral locomotion (NEonset_iloco_ and 5-HTonset_iloco_) in the majority of trials (65.6% and 62.4% of trials for NE and 5-HT, respectively). The frequency of occurrence (before or after onset of locomotion) did not statistically differ for recording trials obtained within the DH, IZ/VH and adjacent WM for either NE or 5-HT (Figure 5E) and there were no statistical differences in the frequency of occurrence between NE and 5-HT. Overall, for all trials and sites, the onset of NE and 5-HT release preceded the onset of ipsilateral locomotion: −6.0 ± 13.4 s and −4.6 ± 13.6 s for NE and 5-HT, respectively. NEonset_iloco_ latencies observed in DH, IZ/VH and WM for trials with best locomotor responses to MLR stimulation, revealed an overall significant difference between locations (one-way ANOVA; *F* = 3.641, *p* = 0.030). *Post hoc* testing showed that NEonset_iloco_ was significantly shorter (*p* = 0.04, Tukey) in the IZ/VH (−10.5 ± 13.5 s, *n* = 37) than in the DH (−2.5 ± 15.1 s, *n* = 28; Figure 5F), but not in the adjacent WM (−3.6 ± 9.8 s, *n* = 28). ANOVA showed no significant differences in 5-HTonset_iloco_ latencies in the DH, IZ/VH and adjacent WM (−2.6 ± 13.5 s, −6.8 ± 14.3 s, −3.7 ± 12.7 s, respectively). As before, a paired samples *t*-test showed that NEonset_iloco_ latency was significantly shorter than 5-HTonset_iloco_ latency in the IZ/VH (*p* = 0.013, *t* = −2.599). Such differences in the latencies of release of NE and 5-HT within the IZ/VH were most noticeable in cases where locomotor activity is delayed relative to the onset of MLR stimulation (Figure 4).

#### Time-To-Peak (TTP)

Monoamine levels in gray and WM reached their peak or nadir values (referred to as the TTP) before termination of MLR stimulation in the majority of trials (78.0% and 65.9% of trials for NE and 5-HT, respectively; Figures 3, 4). This often occurred when locomotor activity waned. Interestingly, NE levels peaked before the end of MLR stimulation significantly more often than 5-HT (McNemar test; *p* = 0.018). Of remaining trials, transmitter levels usually peaked soon after termination of MLR stimulation (12.9% and 16.7% of trials for NE and 5-HT, respectively) and less frequently, continued to increase (or decrease) past the monitoring period (see below). The percentage of trials with continuing release or uptake of NE amounted to 9.1% of the total number of trials (7.6% with increasing levels and 1.5% with decreasing levels). For 5-HT, this amounted to 17.4% of the total number of trials (12.9% with increasing levels and 4.5% with decreasing levels). Such trials were observed in both gray and WM. These results indicate that monoamine levels typically reach their peak (or nadir) in relation to stimulus-evoked locomotor activity and that continued increases in release/uptake past the monitoring period following termination of stimulation and locomotion are relatively rare.

#### Time-To-Decline-to-Baseline (D_50_ and D_100_)

Transmitter levels were continuously monitored for periods of 20–80 s of the termination of MLR stimulation. Periods of altered release usually outlasted the locomotor bouts and the decay of signal could be tens of seconds. By the end of the monitoring period, NE concentrations returned to baseline levels (D_100_) in 90 of 132 trials (68.2%) and at least 50% of baseline levels (D_50_) in 15 more (11.4%). On average, the D_50_ and D_100_ in these trials was 12.9 s ± 9.8 (*n* = 105) and 23.7 s ± 17.6 s (*n* = 90), respectively and did not significantly differ whether NE levels increased or decreased with stimulation. In an additional 15 trials (11.4%), NE levels were returning to baseline levels but had not yet reached D_50_. The remaining 9.1% of trials showed increasing or decreasing NE levels [see “Time-To-Peak (TTP)” Section, above]. For 5-HT, extracellular concentrations had returned to baseline levels by the end of the monitoring period in 73 of 132 trials (55.3%) and to at least 50% of baseline in 10 more (7.6%). On average, the D_50_ and D_100_ in these trials was 11.7 s ± 11.6 (*n* = 83) and 18.2 s ± 14.1 s (*n* = 73), respectively and did not significantly differ whether 5-HT levels increased or decreased with stimulation. In an additional 26 trials (19.7%), 5-HT levels were returning to baseline but had not yet reached D_50_. The remaining 17.4% of trials showed increasing or decreasing 5-HT levels [see “Time-To-Peak (TTP)” Section, above].

### Spinal NE and 5-HT Concentration Changes during MLR Evoked Fictive Locomotion

**Figure 6:**
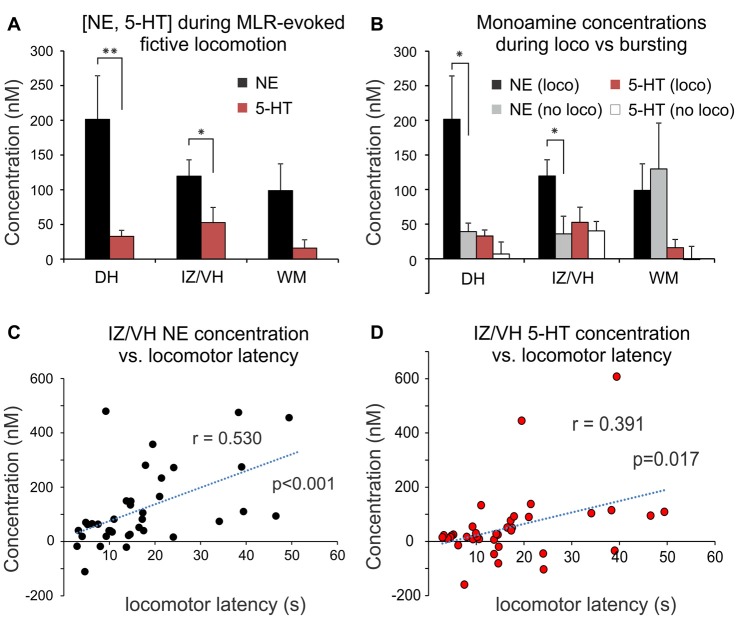
Change in monoaminergic extracellular concentration during MLR-evoked fictive locomotion. **(A)** Peak NE and 5-HT release during MLR-evoked fictive locomotion in DH, IZ/VH and adjacent WM (mean ± SEM). Note that NE release was significantly greater than 5-HT in the gray matter. **(B)** Comparison of peak monoamine concentrations observed with MLR evoked locomotion (loco) vs. no locomotion (bursting) in DH, IZ/VH and adjacent WM. Note that in the DH and IZ/VH, a significantly greater release of NE over baseline was observed in trials with locomotor activity. **(C,D)** Release of NE and 5-HT within the IZ/VH was significantly correlated to the time required to elicit locomotion with MLR stimulation. *p < 0.05, **p < 0.01.

As described above (see “Spatial Patterns of Spinal Monoamine Release with MLR Stimulation” Section), spinal extracellular monoamine concentrations increased above baseline levels more often than decreased in response to MLR stimulation (Figure 5A) and transmitter levels usually peaked during evoked locomotion or shortly after. Overall, for all trials and sites (*n* = 93), NE and 5-HT transmitter concentrations increased above baseline levels during MLR-evoked fictive locomotion by 138.0 ± 232.5 (range: −558.0 to 1271 nM) and 35.6 ± 94.4 nM (range: −159.6 to 607.6 nM), respectively. Since direct stimulation of the locus ceruleus (LC) and raphe magnus in the rat (Hentall et al., 2003, 2006) results in a significantly higher average spinal release of NE than 5-HT, we compared concentrations of spinal 5-HT and NE release during MLR-evoked locomotion. The peak NE and 5-HT concentrations were highly significantly different from each other (*p* < 0.001, *t* = 4.097). NE concentrations observed in DH, IZ/VH and adjacent WM (Figure 6A) were 201.5 ± 331.2, 119.6 ± 142.4 and 98.8 ± 232.5 nM, respectively, and were not significantly different (oneway ANOVA). Similarly, there was no overall differences in 5-HT concentration in the DH, IZ/VH and adjacent WM (32.8 ± 46.1, 52.7 ± 132.5, 15.9 ± 63.3 nM, respectively). However, paired samples *T-test* statistics revealed significant differences between NE and 5-HT in the DH (*p* = 0.010, *t* = 2.787) and IZ/VH (*p* = 0.020, *t* = 2.425) but not in the adjacent WM. Comparing trials with increased release only, NE concentrations averaged 218.8 ± 337.8 (*n* = 26), 139.1 ± 137.8 (*n* = 33) and 136.0 ± 159.2 (*n* = 25) nM for DH, IZ/VH and adjacent WM, respectively. Likewise, in trials with increased 5-HT release, concentrations averaged 45.2 ± 35.6 (*n* = 24), 84.6 ± 130.8 (*n* = 29) and 50.4 ± 54.3 (*n* = 17) nM for DH, IZ/VH and adjacent WM, respectively. No overall differences (oneway ANOVA) were observed for NE in the DH, IZ/VH and adjacent WM concentration and no differences were observed in these locations for 5-HT concentration.

We also examined whether spinal monoamine concentrations differed with the type of MLR evoked activity (Figure 6B). In trials recording from the DH, NE concentration increases were significantly greater (*p* = 0.017, *t* = −2.543) during locomotor (201.5 ± 331.2 nM; *n* = 28) compared to non-locomotor (tonic or phasic excitation lacking reciprocal bursting in antagonists) activity (39.3 ± 42.8 nM; *n* = 12). Similarly, in the IZ/VH, a significantly greater concentration increase of NE was observed (*p* = 0.020, *t* = −2.414) during locomotor (119.6 ± 142.4 nM; *n* = 37) compared to non-locomotor activity (36.0 ± 111.3 nM; *n* = 19). In contrast, although the mean 5-HT concentration increases were greater during locomotion than with non-locomotor activity in both DH [32.7 ± 46.1 nM (*n* = 28) vs. 6.8 ± 60.6 nM (*n* = 12)] and IZ/VH [52.7 ± 132.5 nM (*n* = 37) vs. 40.2 ± 59.4 nM (*n* = 19)], they were not statistically significant. Furthermore, no statistical differences in trials of locomotor (*n* = 28) or non-locomotor activity (*n* = 8) were observed for NE nor for 5-HT release in the adjacent WM. Lastly, in trials recording from the IZ/VH, the concentration of released NE and 5-HT was significantly correlated (Pearson correlation: *r* = 0.530, *p* < 0.001 and *r* = 0.391, *p* = 0.017, respectively) to the time required to elicit locomotion (Figures 6C,D) suggesting that delays in the initiation of locomotion may, in part, be related to the time required to increase monoamine concentrations to appropriate levels. A similar correlation was also noted for NE in the DH (*r* = 0.577, *p* = 0.001) but not for 5-HT. No such correlations were observed for NE and 5-HT release measured in the adjacent WM.

### Reproducibility of Trials and Relationship of Release to Locomotor Intensity/Amplitude

**Figure 7:**
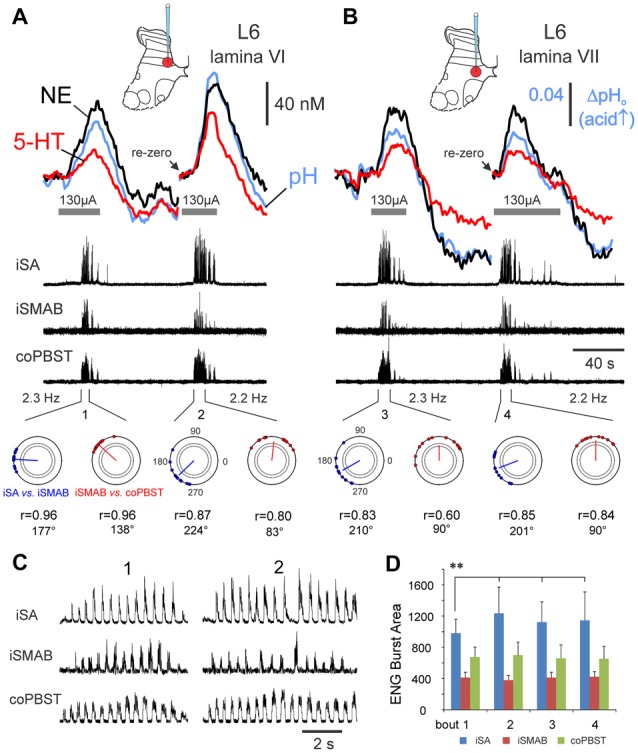
Monoamine release within the spinal cord is modulated in tandem with locomotor activity. Changes in monoamine transmitter and pH levels during repeated trials of MLR-evoked fictive locomotion recorded in medial lamina VI **(A)** and VII **(B)** of the L6 lumbar segment. Release is reproducible when locomotor quality is similar in repeated trials at the same location, as gauged by locomotor frequency, ENG burst area and timing profiles (polar plots—bottom row in **(A,B)**): see similarity in peak concentrations and release profiles for repeated trials in lamina VII (**B**). In contrast, when locomotor quality differs in repeated trials as in **(A)**, the peak concentrations of both NE and 5-HT show positive correlation to the burst area of the ipsilateral flexor nerve (**D**), rather than to locomotor frequency, which does not significantly differ throughout trials (values listed at *bottom* in **(A,B)**). Locomotion is indicated by the recorded ENG activity in *middle traces in* **(A,B)** from various peripheral nerves and in expanded form in **(C)** for bouts 1 and 2. **(D)** ENG locomotor burst area (mean and ± SD, in arbitrary units) for each nerve from bouts 1–4 (*n* = 12, 18, 20 and 18 steps for bouts 1–4, respectively). Note that the averaged locomotor burst area of the iSA in bout 1 was significantly different (**p < 0.01) than for all other bouts. *Horizontal lines* beneath the oxidation current waveform show the period of MLR stimulation (130 μA, 15 Hz, 1 ms duration). Polar plots (*bottom* in **A**,**B**) showing the circular distribution of phase values of 9–12 random steps (*filled circles*) from each bout of locomotion (1–4) and the mean phase values indicated by the angle of the vector originating from the center of the circle. The position on the polar plot indicates the latency of onset of activity for iSA vs. the iSMAB ENGs (*blue*) and for iSMAB vs. contralateral coPBSt ENGs (red). The length of the vector (*r*) ranged from 0.6 to 0.96 and indicated that all nerves were phase-related since the *r* values were greater than the critical Rayleigh’s values (dotted circles inside of the polar plot with *p* values of <0.05 and <0.01). A slight shift in phase value occurred between bouts 1 and 2.

Repeated measurements were made at various spinal locations (*n* = 6) in two animals to assess the reproducibility of transmitter release during trials of MLR evoked locomotion. This is illustrated for two such examples in Figure 7. In Figure 7A, the release of both NE and 5-HT above baseline values in laminae VI increased in tandem with an increased intensity of the ipsilateral flexor ENG as measured by burst area. In this example, the interlimb coordination was also altered. A similar modulation was observed in two additional tested sites. In lamina V, NE release was reduced (from 55.3 nM to 44.8 nM) and 5-HT unchanged (24.2–23.9 nM) in a repeated trial in which the burst area of the ipsilateral extensor was decreased. Additionally, in a trial from lamina VI, 5-HT release was dcreased (from 51.1 nM to 33.8 nM) and NE was relatively unchanged (44.9–43.3 nM) in a repeated trial in which locomotor activity illustrated a reduction in the burst intensity of the ipsilateral flexor. In tests where repeated trials showed comparable locomotor quality and interlimb coordination, the quantity of release was also comparable. This is shown for one such trial from lamina VII (Figure 7B) but was also observed in two additional trials in laminae VII and VIII.

### Extracellular pH Changes during Evoked Locomotion

Local acid changes occur in response to neuronal activity, related to respiratory demands (Chesler and Kaila, 1992; Chesler, 2003) and have been observed in the spinal gray matter during periods of electrical stimulation (Syková and Svoboda, 1990). Similarly, stimulation of the MLR produced a variety of pH changes within the spinal cord, beginning at the same time or slightly earlier to any detectable changes in extracellular monoamine transmitter levels. MLR stimulation produced an initial large acid shift in 102 (77.3%) of 132 trials (Figure 3) which typically lasted the duration of the stimulus. A small number of these trials (*n* = 12) were preceded by a smaller transient base shift (Syková and Svoboda, 1990). In the remaining 30 trials (22.7%), the first response to MLR stimulation was a large base shift. The mean onset latency of acidic changes (with no initial base transient) was 6.3 ± 6.4 s, with a TTP (from onset) of 19.4 ± 18.0 s and a peak of 0.04 ± 0.04 pH units. Small base transients had an onset latency of 5.0 ± 3.2 s, and a TTP of 2.5 ± 1.3 s and a peak of 0.007 ± 0.004 pH units. Consequently, in trials with base transients, acid shifts were delayed with an onset latency of 11.6 ± 6.3 s, a TTP of 43.7 ± 34.4 s and a peak change of 0.03 ± 0.03 pH units. The mean onset latency of large base shifts in trials without preceding acid changes was 8.7 ± 8.2 s, with a TTP of 34.4 ± 26.6 s and a peak change of 0.04 ± 0.05 pH units. Such large initial base changes could often be observed in WM rather than gray. Post-stimulation alkaline shifts were also commonly observed (in 59% of trials) after the initial acidic shifts (Syková and Svoboda, 1990). In contrast, secondary acid shifts were observed in only 30% of trials with initial large base shifts.

### Effect of Monoamine Oxidase and Uptake Inhibitors

**Figure 8:**
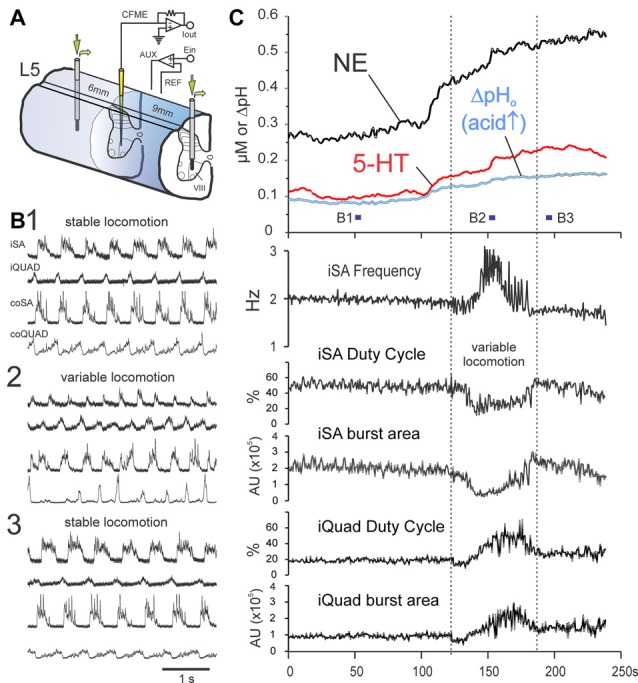
MLR-evoked spinal monoamine release is enhanced by monoamine metabolic and uptake inhibitors and shows modulation during locomotor transition periods. Release of NE and 5-HT in lamina VIII of the L5 segment **(C)** during dialysis of metabolic and uptake inhibitors pargyline (0.5 mM) and desipramine (0.1 mM) through dialysis probes placed 6 and 9 mm distant to the voltammetric electrode (experimental setup illustrated in **(A)**). Trial illustrated shows a 4 min portion, between 4.5 min and 8.5 min, of a 10 min locomotion trial. Locomotor activity for indicated periods in **(C)** are shown in (**B**,**B1,2,3**). At beginning of the illustrated sequence (as represented by locomotor period **B1**), extracellular levels of NE and 5-HT are already high (~275 and 100 nM, respectively) but are reasonably steady while locomotion parameters (frequency, duty cycle and burst area) remain stable **(C)**. Transmitter levels dramatically change just prior to a period of variable locomotion (represented in **B2**) with changes in locomotor frequency and extensor/flexor burst area and burst duration (duty cycle). Following this period, monoamine levels regain a steady-state and locomotion is stabilized **(B3)**. AU: arbitrary units. MLR stimulation parameters: 16 Hz, 160 μA, 1 ms duration pulses.

The effect of monoamine oxidase and uptake inhibitors on MLR-evoked monoamine release was examined in three animals. This is illustrated in Figure 8 for a trial conducted during dialysis of a mixture of 0.5 mM pargyline and 0.1 mM desipramine using two probes separated by 15 mm, with the CFME recording site located between the probes within lamina VIII (L5 segment). In this trial, monoamine release was maintained over the entire stimulation period lasting 600 s (illustrated is a 240 s period occurring between 270 s and 510 s). NE and 5-HT levels continued to increase following the termination of MLR stimulation to peak ~2.5 and 1.5 min post stimulation, respectively. By 22 min, concentrations of both transmitters had declined to ~50% of peak values. Peak concentrations were 685 and 304 nM for NE and 5-HT, respectively. During periods of stable locomotion, NE and 5-HT levels were reasonably steady. However, NE and 5-HT release increased at a much higher rate just prior to a period of variable locomotion which was characterized by shifts in locomotor frequency and changes in extensor/flexor burst area and burst duration (duty cycle). Monoamine levels later steadied once locomotor parameters stabilized. Repeated trials at the same location produced similarly high levels of NE (590–650 nM), 5-HT (~470 nM) and a prolonged TTP (~2 min post stimulation) with D_50_ values between 10 and 20 min. A similar trial conducted in lamina VIII of the L4 segment resulted in comparably lower levels of NE and 5-HT release (375 and 68 nM, respectively), but a prolonged TTP (~13 min post stimulation) was observed (D_50_ and D_100_ not measured). In two other animals, mixtures of the monoamine oxidase inhibitor pargyline (1.3 mM) and the re-uptake inhibitors imipramine (1.5 mM), desipramine (1.6 mM) and buproprion (1.8 mM) were slowly microinjected into the spinal gray matter along each length of one or two adjacent electrode tracks (0.5 mm apart in rostral/caudal direction). Fifteen minutes after microinjection, MLR stimulation trials were commenced. An increase in the extracellular levels of NE (540–900 nM) and 5-HT (up to 250 nM) was observed at sites 1–3 mm distant to the injection sites (laminae VII and VIII of the L4 segment and lamina VIII of the L5 segment; see “Materials and Methods” Section). Levels increased after termination of the MLR stimulus and peaked ~9 min later. In general, these results indicate that monoamine release is enhanced and prolonged by inhibition of monoamine uptake and metabolism.

## Discussion

### General Observations

Changes in the extracellular concentrations of NE and 5-HT were measured at various locations throughout middle and lower lumbar spinal segments during MLR evoked fictive locomotion in the mesencephalic decerebrate cat. Levels were measured relative to the non-locomotor steady state. An increase in the extracellular concentration of NE and 5-HT was most commonly observed during evoked locomotion. Release was widespread: in the IZ/VH, in areas with centrally-activated locomotor neurons (Dai et al., 2005; Noga et al., 2009, 2011); in the DH, where additional neurons are activated by sensory feedback during over-ground locomotion (Dai et al., 2005); and in the WM, likely reflecting, through the process of diffusion, changes in the dynamic equilibrium between release and uptake in adjacent gray matter. Release was rapid on the timescale of seconds and usually began prior to the onset of locomotion, being earliest in the IZ/VH. Transmitter levels were sufficiently high to activate most monoaminergic receptor subtypes expressed by locomotor-activated neurons (Noga et al., 2009, 2011) supporting the idea that extrasynaptic neurotransmission in the spinal cord (Ridet et al., 1993) may be involved in spinal locomotor processes. Highest levels were usually observed during the most vigorous period of locomotion, covaried with net activity in peripheral nerves and were correlated with delays in the initiation of locomotion. NE release was significantly greater in the presence, than absence, of locomotion. Serotonin release within lamina IX covaried with the appearance of ENG locomotor activity (motoneuron firing). Monoamine oxidase and uptake inhibitors also increased the release magnitude, time to peak and time of decline to baseline, implying dynamic control of reuptake. These results demonstrate that spinal monoamine release is modulated on a timescale of seconds, in tandem with centrally-generated locomotion and indicate that MLR-evoked locomotor activity involves concurrent activation of descending monoaminergic and reticulospinal pathways.

### Methodological Considerations/Interpretation of *In Vivo* Voltammetric Findings

By removing background currents that mask faradic (redox) currents (Phillips et al., 2002; Brumley et al., 2007) and applying PCR analysis (Heien et al., 2004; Keithley et al., 2009), we were able to determine changes in the extracellular levels of 5-HT and NE despite the fact that monoaminergic terminal fields are mostly mixed in the spinal cord (Fleetwood-Walker and Coote, 1981; Huisman et al., 1981; Fuxe et al., 1990; Clark and Proudfit, 1991a,b; Doyle and Maxwell, 1991; Fields et al., 1995; Alvarez et al., 1998). Faradic signals were unlikely due to transmitter metabolites and other contaminants since our electrodes were relatively insensitive to them (Hentall et al., 2003). The rapid appearance of the redox signal during evoked locomotion and its usually rapid removal following termination of stimulation (Figures 3, 7) and the fact that the release is enhanced and prolonged by inhibition of monoamine uptake and metabolism (Figure 8) indicates that the signal is due to monoamine overflow rather than their metabolites which take minutes to appear (Michael et al., 1985; Michael and Wightman, 1999). Nafion coating also improves selectivity for the primary amines by reducing sensitivity to monoaminergic metabolites and ascorbic acid (Kuhr and Wightman, 1986; Brazell et al., 1987; Jackson et al., 1995).

Although catecholamines are difficult to differentiate from each other using FCV (Phillips et al., 2002), we maintain that the major component detected in the present experiments is NE, rather than DA: (1) most spinal laminae are densely innervated by descending noradrenergic fibers (Clark and Proudfit, 1991a,b; Grzanna and Fritschy, 1991; Rajaofetra et al., 1992). This is in contrast to their dopaminergic innervation (Skagerberg et al., 1982; Ridet et al., 1992; Holstege et al., 1996). Furthermore, the concentration of DA in the spinal cord is much lower than that of NE (Basbaum et al., 1987); (2) the A11 cell group, the principle source of descending dopaminergic pathways to the spinal cord (Björklund and Skagerberg, 1979; Skagerberg et al., 1982), is absent in the precollicular, postmammillary decerebrate preparation, which spares the A5, A6 (LC) and A7 (Kölliker-Fuse) nuclear groups supplying the noradrenergic innervation of the spinal cord (Westlund et al., 1982); (3) the short stimulus pulse (1 ms) width utilized in the present experiments is more effective for the activation of NE, than DA release (Park et al., 2011); and (4) there are no dopaminergic cell bodies in the lumbar spinal cord (Mouchet et al., 1986; Ridet et al., 1992; Holstege et al., 1996).

### Sites of Release Relative to Location of Locomotor Activated Neurons

In the present series of experiments, monoamine release was examined in mid-to-caudal lumbar segments, where centrally-activated, hindlimb locomotor neurons are most numerous (Dai et al., 2005; Noga et al., 2009, 2011). Stimulation of the MLR evokes the largest dorsal surface potentials in these segments (Noga et al., 1995a), reflecting the depolarization of centrally-activated spinal neurons in laminae VI-X (Jankowska and Noga, 1990; Carr et al., 1994, 1995; Noga et al., 1995a; Huang et al., 2000; Matsuyama et al., 2004). Application of noradrenergic drugs induces walking in spinally injured cats when applied to this area (Chau et al., 1998; Marcoux and Rossignol, 2000; Giroux et al., 2001), indicating that this area contains elements important for the initiation of locomotion. Monoamine release was detected in all areas of the spinal gray matter: DH, IZ and VH. Release in the IZ and VH is likely important in modulating the activity of neurons responsible for the central generation of locomotion. Locomotor activated neurons in these laminae are innervated by descending monoaminergic nerve fibers and possess both serotonergic and noradrenergic receptors implicated in the production of locomotion (Noga et al., 2009, 2011). Release in the DH is likely important for the modulation of additional control elements activated by reflex inputs during the step cycle as seen in treadmill locomotion studies (Dai et al., 2005). Extracellular levels of monoamines are thus dynamically regulated during transitions from non-locomotor to locomotor states and throughout locomotor activity so as to affect the activation of specific subpopulations that produce locomotion and suppress those irrelevant or deleterious to the movement (Harris-Warrick, 1988).

### Comparisons to Previous Studies of Release during Voluntary Locomotion

Spinal monoamine release during treadmill locomotion in the rat has been measured previously by extraction techniques (microdialysis). The temporal resolution in these studies (15 min/sample) necessitates long duration locomotor episodes, resulting in spatial resolutions greater than a millimeter in the diffusional environment of the CNS and increased likelihood of uptake saturation (Lu et al., 1998; Peters and Michael, 1998). This latter consideration makes quantitative interpretation difficult if, at that site, there is an imbalance in the uptake rates of the different transmitters (Lu et al., 1998). Additionally, short-term variations in transmitter release during locomotion (which may still affect second messenger mediated transduction mechanisms) will go undetected with prolonged sampling. Nevertheless, the results from microdialysis measurements of the rat lumbar spinal cord during treadmill locomotion are generally similar to those obtained in the present study, with some important differences. Levels of 5-HT and their metabolites are increased in DH and ventral funiculus WM but decreased in the VH (Gerin et al., 1994, 1995, 2008; Gerin and Privat, 1998). Catecholamine levels are decreased in the DH but are increased in the VH and ventral funiculus WM during locomotion (Gerin et al., 1995, 2011; Gerin and Privat, 1998). Discrepancies may be due to central fatigue under conditions of prolonged activation (Fornal et al., 2006), species differences in uptake kinetics, diffusion rates and the spatial distributions of release sites, differences in baseline levels of monoamines which depend upon the behavioral state (Noga et al., 2004) and differences in the quality of locomotor activity.

### Patterns of Release Relative to Locomotor Activity

#### Increased Monoamine Levels during Stimulation-Evoked Locomotion

To determine whether monamines are released within the spinal cord during MLR-evoked fictive locomotion and to characterize this release with high spatial and temporal resolution, we used FCV methods. Since this method measures release relative to a baseline resting state, our experiments were conducted on mesencephalic decerebrate animals lacking spontaneous locomotor activity with presumably low baseline transmitter levels. The stimulation frequencies used in this study (15–20 Hz) were also within the natural discharge frequencies of raphespinal (Fornal et al., 2006) and ceruleospinal (Aston-Jones et al., 1980) neurons and thus were expected to approximate the release occurring naturally when activated. An increase in monoamine release over background levels was most commonly observed as the first major response to MLR stimulation in DH, IZ and VH. During MLR evoked fictive locomotion, transmitter levels in areas of the IZ/VH where locomotor activated neurons are located (Noga et al., 2009, 2011) increased approximately 150 and 50 nM over baseline, for NE and 5-HT, respectively. These transmitter levels were at concentrations that are physiologically relevant (see “Discussion” Section, below). Interestingly, NE and 5-HT release was correlated to the time required to generate locomotor activity with MLR stimulation. This observation suggests that preparations with slow onset of locomotion had much lower baseline amine levels or were much less excitable (possibly interrelated) and needed higher amine levels to reach sufficient excitation to activate the locomotor network. Additional experiments are warranted to confirm this idea.

#### Decreased Monoamine Levels during and Following Evoked Locomotion

Transmitter levels decreased below steady state levels shortly after the onset of MLR stimulation in a small percentage (~10–20%) of all trials for NE and 5-HT, respectively. Similar responses to stimulation of monoaminergic nuclei have been observed in the spinal cord previously and appear to be highly localized (Hentall et al., 2003, 2006). Additionally, in some locations, following an initial increase in extracellular levels of monoamines at the onset of locomotion, a decrease to baseline or below was observed often as locomotion diminished. Similar findings are observed for some amines sampled from the ventral and DH (see above) of adult rats during prolonged periods of treadmill exercise (Gerin and Privat, 1998; Gerin et al., 2011). Such rapid decreases in transmitter concentration with continuing stimulation likely reflect inhibition of active monoaminergic neurons and removal of transmitters by uptake and clearance mechanisms (Wightman et al., 1988; Jackson et al., 1995) rather than post-translational alterations in rates of uptake or clearance which may take minutes to occur (Frazer et al., 1999). For NE, some LC neurons may not be able to conduct long trains (>20 s of impulses at applied frequencies of 20 Hz (Aston-Jones et al., 1980; see also Hickey et al., 2014) which could account for a failure to maintain extracellular NE levels in some trials of fictive locomotion. For 5-HT, this increase and/or decrease release pattern likely reflects the activity of presumptive serotonergic neurons in the raphe obscurus and pallidus, which increases at or immediately before the onset of treadmill locomotion (Veasey et al., 1995) and which progressively decreases with prolonged locomotion (Fornal et al., 2006). It has been suggested that this is due to the mechanism of “central fatigue” and indicates the strong connection between the firing rate of these neurons and the maintenance of motor performance (Jacobs et al., 2002; Fornal et al., 2006). We favor this as an explanation for the observed profiles of release, since simple modeling of the time-course of extracellular 5-HT indicates that for constantly emitting sources of boutons, there is no spatial arrangement (even with the addition of continuous sinks) that can produce a decline in concentration as that observed here (Hentall et al., 2006). The only alternative explanations would be a diminishment of transmitter release due to vesicle depletion, which is unlikely at the stimulation frequencies used (Hentall et al., 2006), or inhibition of release by auto-receptors or heteroreceptors on terminals of monoaminergic fibers (e.g., Matsumoto et al., 1990; Roberts et al., 1997) by released transmitters. This latter mechanism may be of importance during acute stimulation experiments, considering that high levels of monoamines are observed in highly localized areas of the spinal gray in steady state conditions (Noga et al., 2004).

### Extracellular Monoamines and the Generation of Locomotion—The Role of Volume Transmission in Behavioral State Changes

As observed in the present study, the extracellular concentrations of 5-HT and NE within the lumbar gray matter can increase above baseline to levels that can affect several serotonergic and noradrenergic receptor subtypes (Allgaier et al., 1992; Zoli et al., 1998; Hochman et al., 2001) implicated in the control of locomotion (Marcoux and Rossignol, 2000; Schmidt and Jordan, 2000; Giroux et al., 2001; Hochman et al., 2001; Antri et al., 2003; Liu and Jordan, 2005) and found on locomotor activated spinal neurons (Noga et al., 2009, 2011). Depending on the levels achieved, volume transmission effects would be most selective for higher affinity receptors such as NEα_2_, 5-HT_1A_ and 5-HT_7_ and less so for lower affinity receptors such as NAα_1A_ and 5-HT_2A_ (Hochman et al., 2001; Alexander et al., 2015), which are therefore more likely to be involved in synaptic transmission. Electrical stimulation of the raphe and LC (Hentall et al., 2003, 2006) at rates that exceed those observed at rest and match or exceed those observed during locomotion (Aston-Jones et al., 1980; Veasey et al., 1995; Fornal et al., 2006) also results in a rapid (seconds) increase in extracellular levels of 5-HT and NE within the spinal cord. These spatiotemporal patterns of release are probably paralleled by rapid, local monoaminergic effects on neuronal firing within the spinal cord (e.g., Bras et al., 1990). That the extracellular levels of monoamines are dynamically regulated in widespread regions of the spinal cord at concentrations that are pharmacologically relevant indicates that extrasynaptic or volume neurotransmission (Agnati et al., 1995; Zoli et al., 1998; Fuxe et al., 2007) plays a significant role in the generation and/or modulation of spinal locomotor activity induced by stimulation of the MLR. Alterations in the level of activity within descending monoaminergic pathways and ultimately the extracellular levels of monoamines may be one of the principal mechanisms that mediate changes in “physiological state” (see also, Burke, 1999) so that the effect of descending commands on neural excitability within the spinal cord is fine tuned. In this way, changes in the behavioral state may be the result of neurochemical “resetting” or “remodeling” of interneuronal reflex pathways by volume transmission. By activating the descending monoaminergic system, the MLR can modulate the responsiveness (excitability) of spinal interneurons to input from the reticulospinal pathway (e.g., Jankowska and Noga, 1990; Hammar et al., 2004, 2007) to enable locomotion.

The increase in the concentration of monoamines in the extracellular space is the result of transmitter diffusion out from the synaptic cleft (“spillover”) as well as the direct release into the extracellular space from boutons without opposing synaptic specializations. In the DH, many monoaminergic terminals exhibit little specialization (Maxwell et al., 1983; Marlier et al., 1991; Rajaofetra et al., 1992; Ridet et al., 1992, 1993, 1994; Jankowska et al., 1995, 1997) and a significant proportion of 5-HT receptors and membrane 5-HT transporters are found located remotely from release sites (Ridet et al., 1994; Zhou et al., 1998; Doly et al., 2004). In the IZ/VH, synaptic contacts predominate (Poulat et al., 1992; Ridet et al., 1994; Alvarez et al., 1998), although extrasynaptically-located 5-HT receptors have also been described (Doly et al., 2004).

Extracellular concentrations depend upon many factors related to diffusion, tortuosity and tissue volume fraction and the time-course for changes in extracellular transmitter levels are prolonged in comparison to that for synapses which require diffusion of transmitter over short distances to bind to postsynaptic receptors. On slower time-scales, extracellular volume transmission could account for the tonic depression of monosynaptic group II field potentials in mid-lumbar segments during fictive locomotion (Perreault et al., 1999). For more rapid, differential modulatory effects on group II muscle afferent-evoked field potentials during the flexion and extension phases of locomotion (Perreault et al., 1999), synaptic transmission is likely involved. Both MLR (Garcia-Rill et al., 1983a; Goetz et al., 2016) and raphe neurons (Rasmussen et al., 1986; Jacobs and Fornal, 1995, 1999; Veasey et al., 1995; Fornal et al., 2006) show rhythmically and tonically active neurons during spontaneous locomotion which strengthens the possibility that both modes of transmission from monoaminergic pathways may be utilized at the spinal level.

### Timing of Release Relative to Initiation of Locomotion

Stimulation of the MLR altered spinal NE and 5-HT monoamine release in approximately 8–10 s, respectively. Latencies were most likely related to physiological effects and not a function of the assay method since FCV methods may detect changes in concentrations of monoamines from one scan to the next (<1 s). Release in the IZ/VH occurred at or prior to the onset of bursting and locomotor activity as observed in hindlimb ENGs. NE release was quickest in the IZ/VH and was significantly earlier relative to the onset of bursting or locomotor activity in this location in comparison to the DH or when compared to 5-HT. Latencies for NE or 5-HT release measured in the same locations were often closely related to each other when locomotion was initiated relatively quickly (Figure 3). However, when locomotion was delayed, 5-HT release could be delayed as well (Figure 4). These observations likely account for the significant differences in latencies between NE and 5-HT in this area. They also suggest that the timing of 5-HT release in the VH is more closely tied to motoneuron discharge (as measured with ENG activity) than is NE. Since we did not record interneuronal activity or the activity of motoneurons intracellularly (e.g., locomotor drive potentials—Noga et al., 2003), we cannot determine the timing of the activation of the locomotor network *per se* in relation to the onset of monoamine release. The timing results, however, indicate that 5-HT release in some areas is influenced by factors other than stimulation. Theoretically, 5-HT release could also be affected at the soma or terminal level by autoreceptor or heteroreceptor inhibition (Matsumoto et al., 1990; Roberts et al., 1997), or by inhibitory inputs at the brainstem level. Additionally, 5-HT release may influence the appearance of locomotion (motoneuron discharge) by their action on motoneurons (plateau potentials—see “Discussion” Section, below) or alternatively by actions on reticular neurons (Green and Carpenter, 1985; Di Prisco et al., 1994).

In the rat, monoamine detection following onset of electrical stimulation of the LC or nucleus raphe magnus occurs with a mean latency of approximately 4–4.5 s (Hentall et al., 2003, 2006). Differences in latency between these studies and the present one are likely related to the additional time required for synaptic activation of monoaminergic neurons by MLR projections (as opposed to direct stimulation of the monoaminergic nuclei), the additional axonal conduction time required to reach the spinal cord (Wessendorf et al., 1981; Nakazato, 1987) due to the cat’s larger size, and the differences in stimulation parameters (trains of 50 Hz vs. 20 Hz continuous, 0.2–0.5 ms vs. 1 ms duration for rat and cat, respectively) which affects the time-course/amount of release (Hentall et al., 2003, 2006).

### Functional Aspects of Spinal Monoamine Release during MLR-Evoked Locomotion

It is hypothesized that locomotor pattern and/or frequency is controlled by the activation of descending monoaminergic pathways in parallel with the reticulospinal pathway. By changing the pattern/amount of spinal monoamine release, properties and firing patterns of functionally specific neuron populations including pattern generating circuits to segmental, propriospinal and descending inputs (Bras et al., 1990; Skoog and Noga, 1991; Noga et al., 1992, 1995b; El Manira et al., 1998; Hammar et al., 2004, 2007) may be modulated. Clues as to the functional importance of release at any particular site may be obtained by examining changes in the release profile during repeated locomotor trials that show alterations in locomotor pattern or frequency. This would be predicted on the basis of the effects of monoaminergic drugs on the frequency and pattern of locomotor bursts when administered to the spinal cord (e.g., Barbeau and Rossignol, 1990, 1991; Chau et al., 1998; Brustein and Rossignol, 1999; reviewed by Miles and Sillar, 2011). For example, we observed modulated release of 5-HT and NE over baseline levels near medial gray areas in locomotor trials when flexor and extensor burst amplitudes (pattern) were significantly changed and the timing between sides was modulated, but without concomitant changes in locomotor frequency (Figure 7A). Commissural interneurons in this and adjacent areas are involved in the control of right-left alternation during locomotion (Díaz-Ríos et al., 2007) and could be influenced by monoamines during locomotor activity, since they are innervated by descending reticulospinal neurons (Matsuyama et al., 2004; Szokol et al., 2011) and monoaminergic fibers (Hammar et al., 2004; Noga et al., 2009, 2011). Furthermore, reticulospinal inputs are facilitated by both monoamines, whereas segmental inputs from group II muscle afferents are facilitated or inhibited by 5-HT or NE, respectively (Hammar et al., 2004). This contrasts to that observed in lamina IX where the appearance of locomotor activity was precisely timed to increased levels of 5-HT with or without concomitant changes in the level of NE (Figure 4). Whether this is the result of the induction and/or modulation of plateau potentials in motoneurons (Conway et al., 1988; Hounsgaard et al., 1988) and the subsequent generation of action potentials requires further investigation. MacDonell et al. (2015) have shown that extensor motoneurons become more responsive (primed) during the tonic period of firing immediately prior to the onset of MLR-evoked fictive locomotion. Based on the present results, this neuromodulation could be accomplished by monoamines, which may have significantly more potent neuromodulatory effects on motoneuron excitability than ionotropic actions (Heckman et al., 2009). Additional measurements are needed to determine overall expected segmental and laminar differences (Marcoux and Rossignol, 2000; Liu and Jordan, 2005; Delivet-Mongrain et al., 2008) and the variations between NE and 5-HT release during perturbations that affect not only burst intensity but also the frequency of locomotion. This type of analysis is only possible with the high temporal resolution afforded by FCV.

As discussed elsewhere (Noga et al., 2009, 2011), monoaminergic effects will depend on the cell type and membrane properties, the location and distribution of receptors on individual neurons relative to the site(s) of release, the types/subtypes of receptors and G-proteins expressed in the target cells, the concentrations at synaptic and extrasynaptic sites and therefore the number and type of bound receptors, etc. Since monoamine release is highly dynamic, neuromodulatory effects may also vary temporally during a bout of locomotion. The overall effect of the various monoamines will depend upon the balance and interaction between each neuromodulator acting at the different receptors (Doi and Ramirez, 2010; see also, Beliez et al., 2014). As discussed by Harris-Warrick (2011), the effects of each modulator “may oppose one another, but this may serve to stabilize the modulated state”. Indications that variations in monoamine release may be related to changes in locomotor frequency, extensor/flexor burst area and duty cycle are presented in Figure 8. Here periods of steady state release are associated with stable locomotion.

### Model of Descending Pathways for Initiation of Locomotion

**Figure 9:**
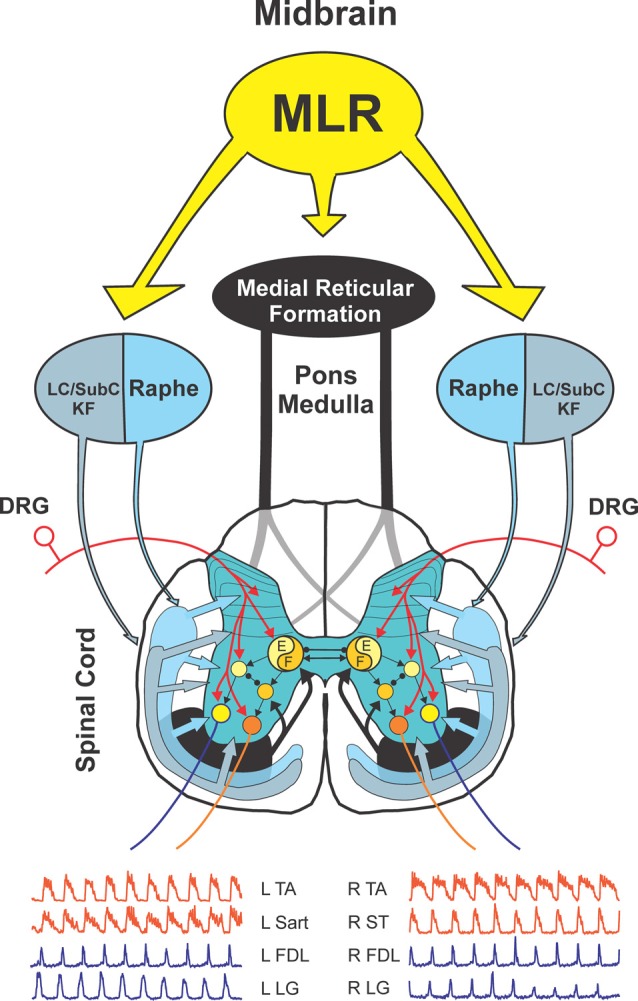
Model of descending locomotor pathways in the cat. Stimulation of the MLR activates reticulospinal neurons which project through the VLF to activate spinal locomotor CPG neurons, in part, by the release of excitatory amino acids. This pathway is considered to comprise the primary “command pathway” for the initiation of locomotion. MLR stimulation also activates in parallel, multiple monoaminergic descending pathways during centrally-generated fictive locomotion. This is evidenced by the rapid, widespread release of NE and 5-HT in the IZ/VH, in areas with centrally-activated locomotor neurons, and in the DH, where additional neurons are activated from afferent feedback from the moving limb during real locomotion. Extracellular monoamine levels are sufficient to activate monoaminergic receptors located on spinal locomotor-activated neurons indicating that extrasynaptic or volume transmission likely plays a significant role in the modulation/generation of locomotor activity. The flexor (F) and extensor (E) components of the locomotor CPG are activated/modulated by descending bilateral reticulospinal and monoaminergic projections as well as by crossed excitatory (▸) and inhibitory (•) segmental projections from the CPG opposite to it. Sensory afferents from skin and muscles innervate spinal neurons in DH, IZ and VH to fine-tune the locomotor step cycle. Details of the flexor and extensor components of the CPG are omitted in order to emphasize general interconnections between them and their target neurons. LC, locus ceruleus; SubC, subceruleus; KF, Kölliker-Fuse; DRG, dorsal root ganglia; L, left; R, right; TA, tibialis anterior, Sart, sartorius, FDL, flexor digitorum longus; LG, lateral gastrocnemius; ST, semitendinosus.

Figure 9 illustrates a modified conceptual model of descending pathways for the production of MLR-evoked fictive locomotion in the decerebrate cat (after Noga et al., 2003). The schematic summarizes the relationships between the various descending pathways, the flexor and extensor components of the spinal locomotor central pattern generator (CPG) and their respective motoneurons for bilateral hindlimb locomotion following stimulation of the MLR. In addition to the reticulospinal “command” pathway, the model incorporates the parallel, bilateral activation of ponto-medullary catecholaminergic and medullary serotonergic pathways and the subsequent spinal release of monoamines. Direct projections from the MLR or its anatomical equivalent to the output neurons of brainstem monoaminergic nuclei have been described (Edwards, 1975; Steeves and Jordan, 1984; Sotnichenko, 1985; Behbehani and Zemlan, 1986). The primary source of spinal monoaminergic innervation originates from cells in these locations (Basbaum and Fields, 1979; Wiklund et al., 1981; Stevens et al., 1982, 1985; Westlund et al., 1982; Nakazato, 1987; Clark and Proudfit, 1991a,b; Jones and Light, 1992). Raphespinal and ceruleospinal neuronal activity increases during spontaneous walking (Rasmussen et al., 1986; Jacobs and Fornal, 1995, 1999; Veasey et al., 1995) further corroborating this idea.

During bilateral hindlimb locomotion induced by unilateral stimulation of the MLR, spinal 5-HT and NE release is likely comparable on both sides. Since the majority of MLR fibers terminate ipsilaterally within the medial reticular formation (Garcia-Rill et al., 1983b; Steeves and Jordan, 1984), and since the majority of catecholaminergic fibers within the LC and dorsolateral pons innervate the ipsilateral spinal cord (Stevens et al., 1985), it is likely that a major portion of catecholamine release *on each side* of the spinal cord come from the terminals of ipsilaterally-projecting pathways. Any potential imbalance on the side opposite to stimulation must be compensated for by bilaterally-projecting catecholamine neurons (especially from the Kölliker-Fuse nucleus) and decussating at each segmental level throughout the cord (Stevens et al., 1982, 1985; Bruinstroop et al., 2012) and/or by MLR projections to the contralateral MLR and Kölliker-Fuse region (Steeves and Jordan, 1984). A similar organization has been described for activation of descending reticulospinal pathways by unilateral stimulation of the MLR where crossed segmental projections likely make up for any potential asymmetry in descending inputs (Noga et al., 2003). MLR projections to the midline raphe magnus and bilateral projections to the post-pyramidal region (Edwards, 1975; Steeves and Jordan, 1984; Sotnichenko, 1985) containing serotonergic neurons projecting to the spinal cord (Jones and Light, 1992), will likely account for bilateral release of 5-HT during locomotion. It is assumed that during spontaneous or voluntary bilateral hindlimb locomotion, MLR activity would be balanced on both sides, as would the descending reticulospinal and monoaminergic input to spinal locomotor centers on either side of the cord.

In the present study, NE release was significantly greater in the presence of organized locomotor activity vs. non-organized or tonic activity (bursting), suggesting that NE may be involved in organizing spinal circuits for coordinated motor output. Both NE and 5-HT release also co-varied in some locations with amplitude modulation of peripheral nerves. A causal relationship between spinal monoamine release and locomotor generation would imply that any left-right asymmetry seen in trials of one-sided locomotion would be due to insufficient and/or disparate activation of descending reticulospinal and monoaminergic neuronal pathways on the affected side. This implies a coupling between locomotor pattern *generation* and monoamine *levels* in cord regions containing locomotor-activated neurons, rather than just a coupling to the stimulation. In our model, the pattern of locomotion will depend upon the amount as well as the spatial and temporal pattern (timing) of release, in addition to the spatial distribution of different functional neuronal populations relative to the site(s) of release, the receptor profiles of the neurons involved and their levels of activity. An insufficiency leading to loss or failure to generate locomotion may result from a substantially reduced signal transmission across *any* potential relay site within the pathway, or possibly be related to some mechanism responsible for central fatigue (Fornal et al., 2006).

With stimulation of the MLR leading up to the formation of locomotor activity, multiple descending pathways are activated resulting in the release of NE and 5-HT within the spinal cord. In many locations within the gray matter, this release occurs within seconds of stimulation, often preceding locomotion and peaking after locomotion ceases. Release is observed in the IZ/VH which is densely innervated by reticulospinal neurons (Peterson et al., 1975; Holstege and Kuypers, 1982; Kausz, 1991) and which contain centrally activated-neurons involved in the generation of hindlimb locomotion (see above), as well as in the DH, where neurons that are activated due to afferent feedback of the moving limb are located (Dai et al., 2005). As predicted from recordings from multiple sites in this study, monoamine release occurs across multiple laminae of the cord during locomotion. Raphe-spinal and ceruleospinal axons typically show extensive collateral branching in the spinal cord which span laminae as well as region (e.g., cervical, thoracic and lumbar; Huisman et al., 1981; Stevens et al., 1982, 1985; Westlund et al., 1982; Nakazato, 1987; Fuxe et al., 1990; Allen and Cechetto, 1994; Fields et al., 1995). Furthermore, recordings from electrophysiologically identified medullary serotonergic neurons show virtually all neurons are activated during treadmill-induced locomotion in cats (Jacobs et al., 2002). This is consistent with the idea that the descending monoaminergic system generally functions as a unit during behavioral activation to provide diffuse descending neuromodulation (see Heckman et al., 2008). Against this background of diffuse neuromodulation, specific spinal inhibitory pathways may function to focus motoneuronal excitability, as needed for specific movements (Heckman et al., 2008). In the present study, single electrode measurements revealed complex, highly unique release patterns from areas of the gray matter in some trials of evoked locomotion. It is possible that these represent unique patterns of release that are not mirrored in other areas during MLR stimulation. Such features could represent variations in the pattern of activation or recruitment (see Chandler et al., 2014; Li et al., 2016) and firing of individual monoaminergic neurons (Jacobs et al., 2002) which change over time (Fornal et al., 2006), and could be influenced by variations in the diffusion distances and/or the geometry of release sites relative to uptake and recording sites (Brumley et al., 2007). Further experiments using simultaneous voltammetric measurements across multiple recording sites would help understand the extent of diffuse neuromodulation during evoked locomotion.

## Author Contributions

BRN and IDH: conceptualization. BRN, RPT, SX, AT and IDH: methodology. BRN, RPT, AP and IDH: investigation. BRN, RPT, SX and AT: formal analysis. BRN and SX: visualization. BRN: writing—original draft. BRN and IH: writing—review and editing, supervision and funding acquisition.

## Conflict of Interest Statement

The authors declare that the research was conducted in the absence of any commercial or financial relationships that could be construed as a potential conflict of interest.
